#  Study of the Protective Effect of *Teucrium polium* L. Extract on Acetaminophen-Induced Hepatotoxicity in Mice 

**Published:** 2013

**Authors:** Hossein Forouzandeh, Mohammad Ebrahim Azemi, Iran Rashidi, Mehdi Goudarzi, Heibatullah Kalantari

**Affiliations:** a *School of Pharmacy, Department of Pharmacology and Toxicology, Ahvaz Jundishapur University of Medical Sciences, Ahvaz, Iran. *; b *School of Pharmacy, Department of Pharmacognosy and Medicinal plants Research Center, Ahvaz Jundishapur University of Medical Sciences, Ahvaz, Iran.*; c *Department of Pathology, School of Medicine, Ahvaz Jundishapur University of Medical Sciences, Ahvaz, Iran. *; d *School of Pharmacy, Department of Pharmacology and Toxicology, Toxicology Research Center and Medicinal plants Research Center, Ahvaz Jundishapur University of Medical Sciences, Ahvaz, Iran. *

**Keywords:** Hepatotoxicity, Acetaminophen, *Teucrium polium* L, Protective

## Abstract

In the present study, protective effect of *Teucrium polium L. (*Labiatae*) *extract on acetaminophen-induced hepatotoxicity was investigated in mice**. **Animals were divided into six groups, each group consist of 8 mice. Group one as the negative control group received normal saline, while group two received only crude extract of *T. polium L. *(500 mg/Kg) for five days and group three as the positive group received acetaminophen (500 mg/Kg). Groups four, five and six received crude extract in doses of 125, 250 and 500 mg/Kg, respectively, and on the fifth day, one hour after the last administration, acetaminophen was given orally (500 mg/Kg). Then on the 6^th^ day, animals were sacrificed, their blood was collected to determine serum enzyme activities of ALT, AST and ALP to measure the serum levels of directed and total bilirubin. The livers were removed for histological examination. The results of this study showed the protective effect in all doses but the most significant protection was observed in doses of 250 and 500 mg/Kg (p < 0.05). Also these findings were supported and confirmed by histological examination.

## Introduction

It is well known that medicinal plants play an important role in health care system and can be called as a main source of new chemical substances with potential therapeutic effects (1). In recent years, there has been a shift towards therapeutic evaluation of herbal products in liver diseases (2). The genus *Teucrium L*. (Labiatae) is very diverse and contains more than 300 species mainly found in the Mediterranean region (3). *Teucrium polium L. is a wild-growing flowering plant, *which belongs to Labiatae family (4). This plant has been used in folk medicine for various purposes such as anti-inflammatory, anti-bacterial, anti-pyretic, anti-spasmodic, anti-hypertensive and anti-hyperlipedemic (5, 6). Furthermore, the plant possesses hypoglycemic, insulinotropic, diuretic, diaphoretic, tonic, cholagogic and antioxidant properties (8-11). Acetaminophen is a widely used nonprescription analgesic and antipyretic drug that has a very low rate of liver toxicity at normal therapeutic doses, however, it causes hepatic and renal injuries in humans and experimental animals when administered in high doses (12-14 ). Liver, as a major vital organ, metabolizes acetaminophen in the form of glucuronidated and sulfated product and the subsequent metabolite is excreted by urine (15), but small fraction metabolized by cytochrome P_450_ to a highly reactive free radical, *n*-acetyl-*p*-benzoquinone imine (NAPQI) (16). This metabolite is a strong electrophile oxidizing agent normally detoxified by reduced glutathione (GSH) in the liver (17). However, after acetaminophen overdose, the glucuronidation and sulfation pathways become saturated, and more acetaminophen becomes available for activation by the cytochrome P_450_, which produces a large amount of NAPQI leading to rapid depletion of hepatic GSH levels. Subsequently, NAPQI metabolite binds covalently to cell macromolecules that results in cell damage or cell death (18, 19). The aim of this study was to evaluate the hepatoprotective activities of hydro alcoholic extract of *T. polium *on acetaminophen-induced hepatotoxicity.

## Experimental

*Animals*

Studies were carried out using male ICR mice (6-8 weeks old, 25-30 g), obtained from Animal house of Ahvaz Jundishapur University of Medical Science, Iran. Mice were kept in polycarbonate cages under standard condition (temperature 25 ± 2°C) with 12 h light/dark cycle. They were provided with standard pellet diet and free access to drinking water *ad libitum. *The animals were acclimatized to the environment for a week before the commencement of experiment. The investigation was performed according to the Local Animal Ethics Committee guidelines for the use of experimental animals.

*Chemicals*

All the chemicals were of analytical grade. Solvents were purchased from Merck (Darmstadt, Germany). The acetaminophen powder was purchased from Darou pakhsh Company (Iran). *Preparation of plant extract*

Plant was collected from Larestan region, Iran in April 2008 and shade-dried. The plants were identified at the Herbarium of Department of Pharmacognosy, School of Pharmacy, Ahvaz, Iran, where the voucher specimens were preserved (number voucher: T-0157). The whole plant was crushed into small pieces and soaked in an 80% aqueous-ethanol solution in a large container for 3 days with occasional shaking. The extract was filtered through a clean cotton cloth and the filtrate was dried by using a rotary evaporator at 40°C. The extract yield was 16% w/w (19).

*Study design*

Plant extract was dissolved in normal saline before the administration to mice. Acetaminophen was first dissolved in normal saline at 70°C, and then cooled to 37°C for administration; it was administered orally in a single dose of 500 mg/Kg. Mice were divided randomly into six groups, each of which consisted of eight animals. All mice were treated orally for five consecutive days. Group one as the negative control group received normal saline (10 mL/Kg), while group two received only crude extract of *T. polium L. *(500 mg/Kg) for five days and group three as the positive group received acetaminophen (500 mg/Kg) on the fifth day. Groups four, five and six received crude extract during five days in doses of 125, 250 and 500 mg/Kg, respectively and on the fifth day, acetaminophen was administered (500 mg/Kg) one hour after the last administration of the crude extract. Then, on the 6^th^ day, animals were sacrificed and their blood was collected to estimate ALT, AST and ALP, direct and total bilirubin. Liver was removed and kept in 10% formalin solution for histopathological examination.

*Biochemical assays*

The blood samples were allowed to clot for 40 min at room temperature. Serum was separated by centrifugation at 2500 rpm at 30°C for 15 min. Activities of aspartate aminotransferase (AST) and alanine aminotransferase (ALT) in serum were determined employing the method of Reitman and Frankel. In this procedure, transaminase reacts with 2, 4-dinitrophenyl-hydrazones reagent and produces color complex that is proportional to the AST and ALT concentrations (21). Alkaline phosphatase (ALP) was estimated according to king (22), while serum levels of direct and total bilirubin were determined according to the method of Watson and Rogers. In this method, the conventional diazo reagent was added to plasma, and then the color densities were determined (23).

*Histopathological assessments*

The liver specimens were fixed in 10% neutral buffered-formalin and then, Liver tissues were dehydrated with a sequence of ethanol solutions, embedded in paraffin, cut into 5 μm sections, stained with hematoxylin and eosin dye (H and E stain) and observed under a photomicroscope (24). The observed morphological changes included cell necrosis, fatty change and the infiltration of lymphocytes and Kupffer cells.

*Statistical analysis*

Statistical analysis was performed using the statistical package SPSS 16.0 for Windows. The results were expressed as mean ± SD. One-way ANOVA followed by Tukey post-test were applied for statistical analysis with the level of significance set at p < 0.05.

## Results

It is clear that an increase in serum levels of AST, ALT, ALP and bilirubin concentrations in blood indirectly reflects the failure of liver function due to the acetaminophen-induced hepatotoxicity. The effects of pre-treatment with different doses of *T. polium *extract (125, 250 and 500 mg/Kg) on serum levels of liver enzymes and bilirubin in acetaminophen-induced hepatotoxicity are shown in Tables 1 and 2.

**Table 1:** Effects of the pretreatment with *Teucrium polium *L. extract on the serum activities of AST, ALT and ALP in acetaminophen-induced hepatotoxicity

**Groups**	**AST (U/L)**	**ALT (U/L)**	**ALP (U/L)**
1. normal saline	73.87 ± 20.08 ^b^	63.25 ± 8.92 ^b^	88.62 ± 10.79^b^
2. *T. polium *(500 mg/Kg)	81.75 ± 21.75^b^	61.62 ± 8.65^b^	95.62 ± 13.98 ^b^
3. Acetaminophen (500 mg/Kg)	848.50 ± 38.26^a^	683.50 ± 42.63^a,^	171.62 ± 16.87 ^a^
4. *T. polium *125 mg/Kg + Acetaminophen	702.12 ± 24.58^a,b^	547.00 ± 41.70 ^a,b^	128.50 ± 15.08 ^a b^
5. *T. polium*250 mg/Kg + Acetaminophen	406.00 ± 20.52^a,b^	338.75 ± 45.24 ^a,b^	101.00 ± 16.97 ^b^
6. *T. polium*500 mg/Kg + Acetaminophen	441.50 ± 112.68^a,b^	354.00 ± 43.98 ^a^,^b^	96.50 ± 12.85 ^b^

**Table 2:** Effects of the pretreatment with *Teucrium polium *L. extract on the serum levels of direct and total bilirubin in acetaminophen-induced hepatotoxicity

**Groups**	**Direct bilirubin**	**Total bilirubin**
**1**. normal saline	.3100 ± 06437	.4925 ± .12361
**2**. *T. polium *(500 mg/Kg)	.3438 ± .08450	.5112 ±.14025
**3**. Acetaminophen (500 mg/Kg)	.4225 ± .08940	.6075 ±.18211
**4**. *T. polium *125 mg/Kg + Acetaminophen	.4488 ± .09658	.5762 ±.13511
**5**. *T. polium *250 mg/Kg + Acetaminophen	.3900 ± .10981	.5925 ±.15360
**6**. *T. polium *500 mg/Kg + Acetaminophen	.3712 ± .11319	.5412 ±.15797

Animals in group 1 received normal saline solution, whereas animals in group 2 received T. *polium L. *extract (500 mg/Kg) for 5 days. Group 3 received acetaminophen on the fifth day. The mice in groups 4, 5 and 6 were pretreated with T. *polium L. *extract (125, 250 and 500 mg/Kg, po, respectively) once daily for five consecutive days. One hour after the final treatment, the mice were treated with acetaminophen (500 mg/Kg, po). Each value represents the mean ± SD for eight mice. ^a^: Significantly different from the control (p < 0.05). ^b^: Significantly different from acetaminophen group ( p < 0.05).

**Figure 1:**
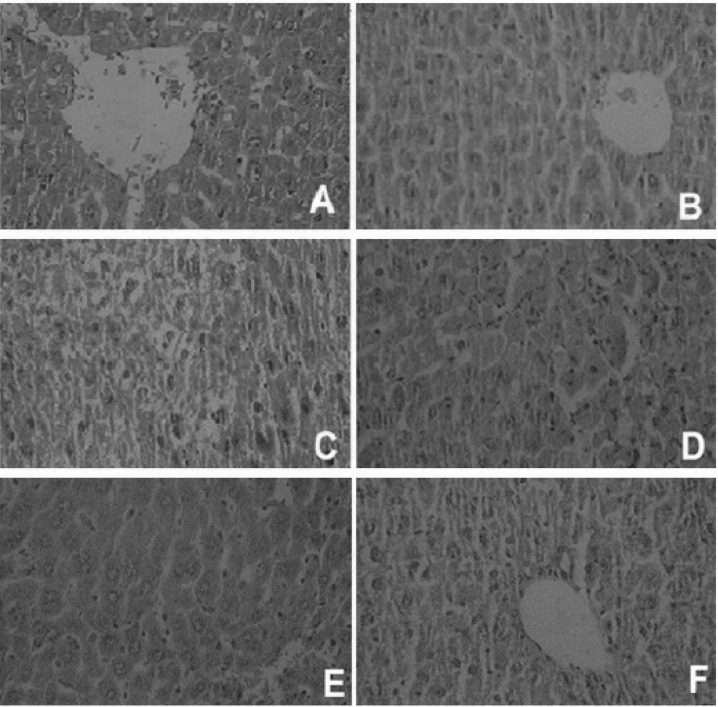
Histopathological observations (liver sections stained with Hematoxylin and Eosin, magnification x 100) showing the effects of *Teucrium polium *extract on acetaminophen-induced histopathological changes in mouse liver. (A) Normal, (B) T. *polium L. *extract (500 mg/Kg) shows normal hepatic architecture with distinct hepatic cells, sinusoidal spaces and a central vein; (C) acetaminophen-treated group shows severe centrilobular hepatic necrosis, fatty changes, ballooning degeneration, and infiltrating lymphocytes; (D), (E) and (F) are acetaminophen groups pre-treated with 125, 250 and 500 mg/Kg of *T. polium L *extract, respectively. D shows milder degree of hepatocyte necrosis, fatty changes, ballooning degeneration, and infiltrating lymphocytes. In pictures E and F, only mild inflammation and lymphocyte infiltration are observed

Data showed that 24 h after the acetaminophen administration mice developed significant hepatocellular damage as it was evident from a significant increase in the serum activities of ALT, AST and ALP (p < 0.05) when compared with the negative control group (Table 1). The positive control group showed slightly (not significant) increase in serum direct and total bilirubin concentration, as shown in Table 2. Pretreatment of mice with 125, 250 and 500 mg/Kg of hydroalcoholic extracts of *T. polium *exhibited a significant (p *< *0*.*05) reduction in the levels of AST, ALT and ALP as compared with the positive control group (Table 1). Direct and total bilirubin concentration, elevated by acetaminophen, exhibited a slight decrease in response to the pre-treatment with the plant extract (Table 2). The histopathological study of liver in the negative control group and the second group with only crude extract showed a normal hepatic architecture with distinct hepatic cells, sinusoidal spaces and a central vein (Figures 1A and 1B).

Acetaminophen-intoxicated group exhibited severe histopathological changes, such as centrilobular hepatic necrosis, fatty changes, ballooning degeneration, and infiltrating lymphocytes (Figure 1C). The group that received 125 mg/Kg of *T. polium *extract showed partial protection of hepatocytes (Figure 1D), while pretreatment with doses of 250 and 500 mg/Kg of hydro alcoholic extract showed best results and prevented these histopathological changes associated with the hepatotoxicity induced by acetaminophen. Results of groups that received 250 mg/Kg and 500 mg/Kg of the extract *of T. polium *are shown in Figure 1E and Figure 1F.

## Discussion

Liver is one of the largest organs in human body and the major site for metabolism and excretion (25). It has a wide range of functions, including detoxification, protein synthesis, and production of biochemical’s necessary for digestion (26). Liver diseases have become one of the major causes of morbidity and mortality in human all over the world and hepatotoxicity due to drugs appears to be the most common contributing factor (27). Protection against acetaminophen induced toxicity has been used as a test for a potential hepatoprotective agent by several investigators (28-31).

Acetaminophen, as an analgesic and antipyretic drug, is readily available without prescription. In therapeutic doses, acetaminophen is well tolerated; side effects and interaction with other drugs are usually not observed. However, overdose of acetaminophen causes acute centrilobular hepatic necrosis in humans and experimental animals (32, 33). The rise in serum levels of transaminases (AST and ALT) and ALP have been attributed to the damaged structural integrity of the liver, because these are normally located in the cytoplasm and are released into the circulation after cellular damage (34). The elevation in the levels of bilirubin has been reported in acetaminophen-induced hepatotoxicity. Bilirubin, an endogenous organic anion binds reversibly to albumin and it is transported to the liver, and then conjugated with glucuronic acid and excreted in the bile. In hepatobiliary disease, bilirubin concentration exceeds the upper limits of normal (35, 36). Since hepatic damage induced by acetaminophen is mediated by its free radical metabolites (16-18), antioxidant activity or inhibition of the generation of free radicals is important in the protection against acetaminophen-induced liver injury (37). It has been reported that *T. polium *possess profound anti-inflammatory and antioxidant activity (10, 11, 38). Furthermore, *T. polium *extract enhances intracellular glutathione levels by promoting the glutathione biosynthetic pathway (39). These properties motivate us to study its hepatoprotectivity in acetaminophen-induced liver toxicity.

Panovska *et al. *investigated hepatoprotective activity of the ethyl acetate extract of *Teucrium polium *L. against CCl4-induced liver damage. They reported that intraperitoneal injection of *T. polium *extract for 7 days resulted in restoration of liver damage to the normal state (40).

Our results provided strong evidence that *T. polium *extract significantly inhibited the acute liver toxicity induced by high doses of acetaminophen in mice, as shown by a decrease in serum liver enzyme activities (AST, ALT and ALP) and bilirubin concentrations (Tables 1 and 2). Moreover, the liver morphology and histopathology findings confirm the protective activity of this extract against the acetaminophen-induced liver damage as it is evident by the reversal of centrilobular necrosis, fatty changes (steatosis) and scattered lymphocytes infiltrate in hepatic parenchyma by *T. polium *administration. Thus, as shown in Figures 1E and 1F, only mild inflammation and lymphocyte infiltration were observed. Although this protective effect was dose-dependent, there was no significant difference between doses of 250 and 500 mg/Kg of *T. polium *extract (Table 1). Despite the fact that *T. polium *extract significantly reduced ALT and AST levels in groups 4, 5 and 6, it can’t completely restore these biochemical parameters to the normal values. Moreover, group two that received only crude extract of *T. polium L *(500 mg/Kg, once daily, for 5 days) did not have any significant differences with negative control group based on biochemical parameters (AST, ALT , ALP and bilirubin) and histopathological findings and did not cause liver damage. Similarly, in the study of Iriadam *et al*, oral administration of *T. polium L *(82 mg/Kg) did not cause any adverse effect on liver (39).

In conclusion, this herbal extract had potential protective effect in all doses but the most significant protection was observed in doses of 250 and 500 mg/Kg (p < 0.05). In addition, these findings were supported and confirmed by histological examination. Further studies are required to isolate and purify the bioconstituents involved in hepatoprotection of this plant as well as to confirm the mechanisms responsible for hepatoprotective activity.
